# Supplementary material for: Preparation and pH Controlled Release of Fe3O4/Anthocyanin Magnetic Biocomposites
Source: Polymers (Basel). 2019 Dec 12;11(12):2077. doi: 10.3390/polym11122077 (PMC6960501; doi:10.3390/polym11122077)
Supplement: Supplementary file 1 [file polymers-11-02077-s001.pdf]

Supporting information

for

Preparation and pH controlled release of Fe_3O_4/anthocyanin magnetic biocomposites

Xizhi Jiang [1,2,3], Qingbao Guan [4], Min Feng [2,3], Mengyang Wang [2,3], Nina Yan[2,3], Min Wang[2,3], Lei Xu [2,3,] and Zhongzheng Gui [1,]**

[1] School of Biotechnology, Jiangsu University of Science and Technology, *Zhenjiang 212018, Jiangsu, China*

[2] Institute of Agricultural Facilities and Equipment, Jiangsu Academy of Agricultural Sciences, *Nanjing 210014, Jiangsu, China*

[3] Key Laboratory for Protected Agricultural Engineering in the Middle and Lower Reaches of Yangtze River, Ministry of Agriculture and Rural Affairs, *Nanjing 210014, Jiangsu, China*

[4] State Key Laboratory for Modification of Chemical Fibers and Polymer Materials, International Joint Laboratory for Advanced Fiber and Low-dimension Materials, College of Materials Science and Engineering, Donghua University, *Shanghai, 201620, China*

** Corresponding Authors*

E-mail: xulei@jaas.ac.cn (L. Xu); srizzgui@hotmail.com (Z. Gui)

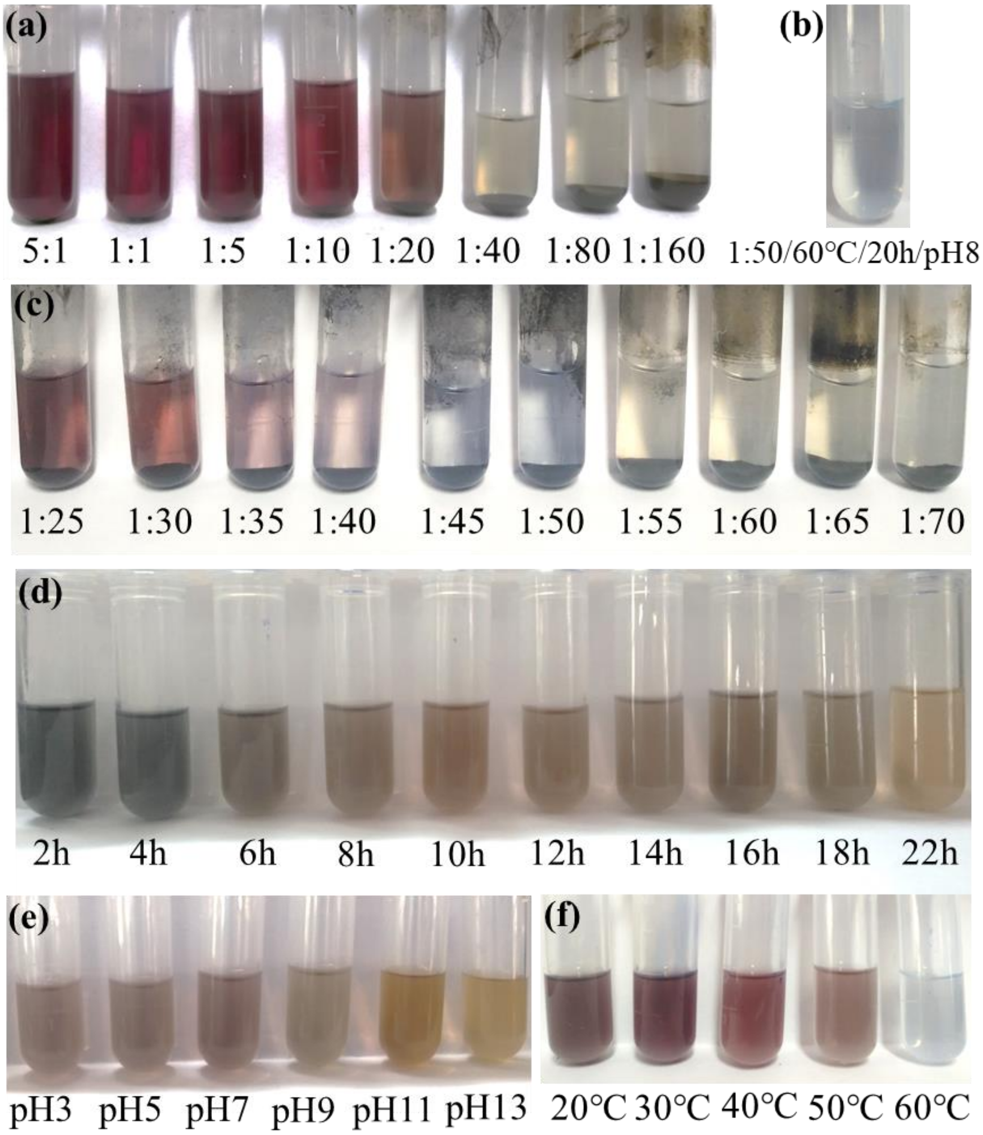

Figure S1. The images of optimized synthesis conditions of Fe_3O_4/anthocyanin magnetic biocomposite. (a) Anthocyanin and Fe_3O_4 with different mass ratios of 5:1, 1:1, 1:5, 1:10, 1:20, 1:40, 1:80; (b) The optimum synthesis conditions of Fe_3O_4/anthocyanin magnetic biocomposite; (c) Anthocyanin and Fe_3O_4 with different mass ratios of 1:25, 1:30, 1:35, 1:40, 1:45, 1:50, 1:55, 1:60, 1:65, 1:70; (d) Different reaction times of 2, 4, 6, 8, 10, 12, 14, 16, 18, 22 h; (e) Different pH of 3, 5, 7, 9, 11, 13; (f) Different reaction temperatures of 20, 30, 40, 50, 60 °C.

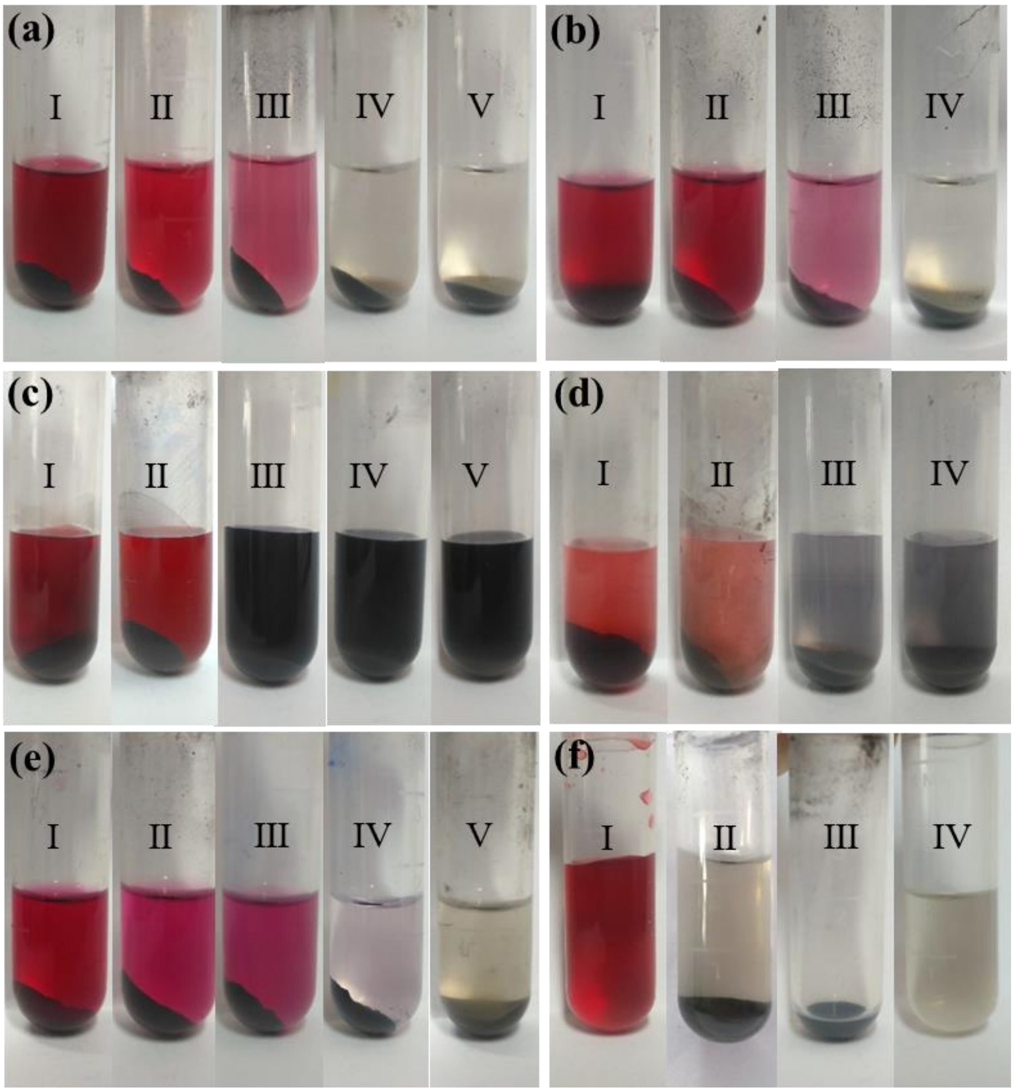

Figure S2. The images of release conditions of anthocyanin. (a) Primary release in different methanol solutions (pH 1.0, 1.6, 2.0, 3.0 and original methanol solution); (b) Secondary release in different methanol solutions (pH 1.0, 1.6, 2.0, 3.0); (c) Primary release in different deionized water solutions (pH 1.0, 1.6, 2.0, 3.0 and deionized water solution); (d) Secondary release in different deionized water solutions (pH of 1.0, 1.6, 2.0, 3.0); (e) Primary release in different ethanol solutions (pH 1.0, 1.6, 2.0, 3.0 and original ethanol solution); (f) Anthocyanin solution, Fe_3O_4/anthocyanin magnetic biocomposites, separated Fe_3O_4/anthocyanin solid, supernatant, respectively.

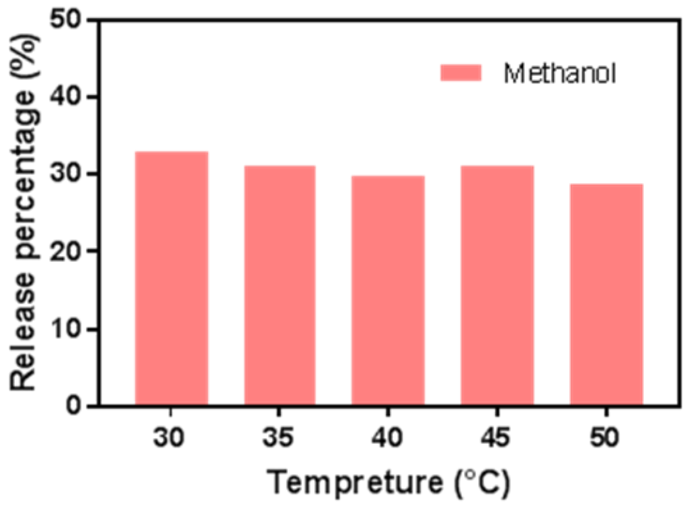

Figure S3. Release percentages of anthocyanin from Fe_3O_4/anthocyanin magnetic biocomposites at different temperatures

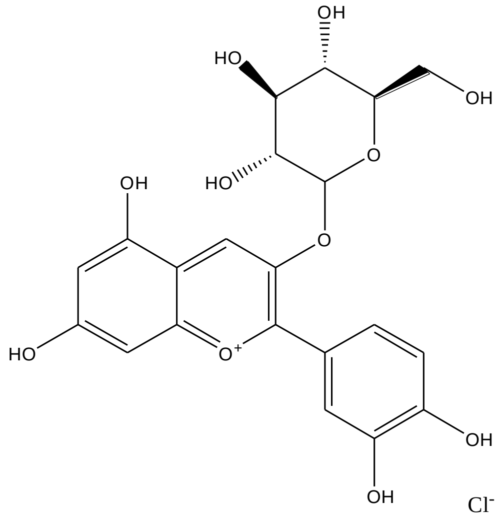

Figure S4. The structure of cyanidin-3-O-glucoside standard
